# The Complexity of Biomechanics Causing Primary Blast-Induced Traumatic Brain Injury: A Review of Potential Mechanisms

**DOI:** 10.3389/fneur.2015.00221

**Published:** 2015-10-19

**Authors:** Amy Courtney, Michael Courtney

**Affiliations:** ^1^Exponent Engineering and Scientific Consulting, Philadelphia, PA, USA; ^2^BTG Research, Baton Rouge, LA, USA

**Keywords:** blast injury, traumatic brain injury, blast wave transmission, thoracic mechanism

## Abstract

Primary blast-induced traumatic brain injury (bTBI) is a prevalent battlefield injury in recent conflicts, yet biomechanical mechanisms of bTBI remain unclear. Elucidating specific biomechanical mechanisms is essential to developing animal models for testing candidate therapies and for improving protective equipment. Three hypothetical mechanisms of primary bTBI have received the most attention. Because translational and rotational head accelerations are primary contributors to TBI from non-penetrating blunt force head trauma, the acceleration hypothesis suggests that blast-induced head accelerations may cause bTBI. The hypothesis of direct cranial transmission suggests that a pressure transient traverses the skull into the brain and directly injures brain tissue. The thoracic hypothesis of bTBI suggests that some combination of a pressure transient reaching the brain via the thorax and a vagally mediated reflex result in bTBI. These three mechanisms may not be mutually exclusive, and quantifying exposure thresholds (for blasts of a given duration) is essential for determining which mechanisms may be contributing for a level of blast exposure. Progress has been hindered by experimental designs, which do not effectively expose animal models to a single mechanism and by over-reliance on poorly validated computational models. The path forward should be predictive validation of computational models by quantitative confirmation with blast experiments in animal models, human cadavers, and biofidelic human surrogates over a range of relevant blast magnitudes and durations coupled with experimental designs, which isolate a single injury mechanism.

## Introduction and Background

Blast-induced traumatic brain injury (bTBI) is not a new battlefield injury^1^. Mott (1) published a discussion in which both bTBI (which Mott referred to as “shell shock”) and post-traumatic stress disorder (PTSD) (which Mott termed “psychic trauma”) are discussed as distinct possible outcomes of blast exposure. Beginning in the late 1990s and increasingly since, bTBI has gained military and public prominence in the U.S. as an injury that needs to be prevented as well as treated. Between 2000 and 2014, more than 300,000 American soldiers were diagnosed with traumatic brain injury of any type (2)^2^. As of March, 2014, more than 80% of these diagnoses were classified mild TBI^3^. The sharp increase in mild TBI beginning in 2006, as well as field data indicating that 50–80% of battlefield injuries have been due to blast exposure, are consistent with the increase in mild TBI cases resulting from blast exposure (3, 4). Effective solutions are needed for military, humanitarian, and economic reasons.

Blast injury has long been classified in a way that is broadly consistent with expected external mechanisms, though decades ago the focus was on potential exposure to nuclear blasts and underwater blasts (5). Primary blast injury refers to injury that is caused by exposure to a blast wave itself. Secondary blast injury refers to blunt or penetrating trauma sustained when material is propelled by a blast and strikes the body. Tertiary blast injury results when the body itself is set in motion and strikes the ground, a structure, or some other object to result in injury. Secondary and tertiary injuries are similar in some ways to blunt trauma due to other mechanisms. The distinction between secondary and tertiary blast injury is somewhat academic according to Newton’s third law of motion. However, it is a useful distinction for those developing military vehicles and equipment, for example, as they work to minimize the risk of injury in a blast event.

Quaternary blast injury is a catch-all for other injury mechanisms attributable to an explosion, such as crush injuries, burns, and the exacerbation of chronic ailments, such as angina and hypertension. Some have proposed a quinary classification to distinguish a hyperinflammatory response observed in some individuals following blast exposure. This injury is hypothesized to be a reaction to exposure to toxic materials released in an explosion (6). However, a quinary classification does not often appear in the literature as a distinct category.

The U.S. government has made large expenditures for traumatic brain injury treatment and research. In 2007, $900 Million was allocated to the Department of Defense in a single appropriations act – $600 Million for treatment of TBI and PTSD and $300 Million for research^4^. In 2011–2013, the National Institutes of Health spent an additional $80 Million to $90 Million per year on traumatic brain injury research (not only blast injury)^5^. Proposed mechanisms for primary bTBI are being investigated experimentally, and numerical models have been developed to facilitate the prediction of experimental results, with the goal of elucidating injury mechanisms and thresholds and speeding the development of preventive measures.

However, although research expenditures and the resulting body of information have ballooned in recent years, results of laboratory experiments are often difficult to compare across studies or to place in the context of battlefield threats. There are important gaps in research and lack of access to field data that need to be addressed in order to correctly interpret and apply the results of recent research. For example, reports of laboratory studies of bTBI in animal models sometimes do not sufficiently characterize the essential features of the blast exposure, including peak pressure (and whether the reported pressure is incident or reflected) and positive pulse duration at the location of the exposed animal. If a compressed gas shock tube is used that might impart a second insult due to gas expansion, this second insult is rarely, if ever, quantified. Clinically, the distribution of actual exposures to personnel is not readily available. A recent review noted that “a limitation of nearly all the studies evaluated by the committee was inadequate information about the exposure to blast” (6). Moreover, even the most sophisticated numerical models lack predictive validation and are further limited because reliable input values for material properties of the highly viscoelastic skull and brain tissues at blast strain rates are not available.

The purpose of this paper is to review experiments and modeling efforts relevant to three broad biomechanical mechanisms for primary TBI. This paper does not review the epidemiology of primary bTBI or review the ample evidence of brain injuries or exacerbations that result from physiological responses over time after the blast exposure (7–9). The latter important topic is an active area of research and potential therapeutic intervention (10–12). This review is intended to remind of important principles and demonstrated results as well as limitations in experimental and modeling efforts, limitations that might be avoided in future efforts with cognizant planning and adherence to the scientific method. It is also meant to inspire careful discussion, testable hypotheses, and rigorous experiments.

## Primary Blast Injury

As stated above, primary blast injury refers to injury that is caused by direct exposure to the shock and pressure of a blast wave itself. Once thought to be restricted to gas-containing organs, such as the lungs and the intestines, primary blast injury has been shown to manifest in additional ways, including primary bTBI. Though the concept of “shell shock” has been in the literature for a century, there has been renewed debate over whether a blast wave alone can injure the brain. In the 1990s, Dr. Ibolja Cernak and colleagues, treating hundreds of patients injured by blast at the Military Medical Academy in Belgrade, documented a pattern of injury associated with blast exposure that could not be attributed to secondary or tertiary mechanisms (13). As described by Battacharjee (14), clinical observations of symptoms following primary blast exposure motivated the development of hypotheses and the design of experiments. Experiments yielded data showing it is possible for blast waves alone to injure the central nervous system separately from penetrating injury or blunt trauma (15, 16). As previously reviewed, results of numerous-independent blast and ballistic studies support the general hypothesis that primary exposure to blast waves can result in tissue damage [Ref. (17, 18) and references therein].

As scientific debate continued, military conflicts involved a marked increase in the use of improvised explosive devices. Improvements in tactics, armor, and battlefield medicine reduced deaths from penetrating injuries that were commonly fatal in earlier conflicts, such as the Vietnam War (6); nevertheless, the higher number of blast exposures resulted in significant morbidity, so that the ratio of U.S. warfighters wounded to killed in action was >9:1 in recent conflicts compared to ratios ranging from 2:1 to 4:1 in prior conflicts (19). Among the survivable injuries in recent conflicts, a distinct pattern of brain injury was recognized among soldiers exposed to blast. Most diagnoses have been of mild bTBI.

Symptoms of mild bTBI are sometimes similar to concussion from sports injuries or other blunt trauma; cognitive and emotional deficits observed in these patients are sometimes similar to, or may instead be due to PTSD. This apparent overlap contributed to debate whether bTBI is a unique injury. Elder et al. (20) recently published a review of clinical and animal studies related to this issue. They point out the clinical difficulties in distinguishing these diagnoses and also the role differing clinical criteria for mild bTBI may play in the increased number of diagnoses. However, based on their review of clinical and laboratory data, they concluded that mild bTBI can induce PTSD-related behavioral traits “in the absence of a psychological stressor,” and that a variety of biochemical, pathological, and physiological effects on the nervous system have been observed in rodent models of bTBI. From the biomechanical perspective of this review, because of the lack of specific information regarding the circumstances of each exposure in humans, it is still unclear what fraction of bTBI is due to primary versus secondary or tertiary causes, or some combination thereof. However, certain functional and behavioral symptoms, and the absence of visible trauma, are consistent with primary blast injury being a unique injury.

A variety of experiments in larger animal models also support the uniqueness of primary bTBI as a physical injury. Bauman et al. (21) reproduced pathophysiological characteristics of bTBI in 40–50 kg swine outfitted with lead and foam-lined vests and exposed to explosive blast without the possibility of secondary or tertiary mechanisms. Experiments in large diameter blast tubes and simulated vehicle and building interiors illustrated that complex blast waveforms may effectively result in multiple insults from a single blast in theater. In the blast tube, peak pressures in the brain were recorded to be approximately half of the pressure measured near, but external to the head. However, the reported injuries could not be considered along with absolute levels of exposure, which were not reported. While these experiments are a clear example of primary bTBI, they do not address the specific mechanism(s) of injury, and the complex loading environments, while realistic to some exposures, do not quantify threshold levels.

A recent experiment by Lu et al. (22) provided information regarding overall exposure thresholds for bTBI and related cognitive deficits in cynomolgus macaques (*Macaca fascicularis*). Anesthetized subjects were exposed to a single blast at 80 kPa overpressure, a single blast at 200 kPa overpressure, or two blasts (3 days apart) at 80 kPa overpressure, each having a positive duration of approximately 7–15 ms. Blast loading was achieved by a free-field detonation of 2,4,6-trinitrotoluene (TNT), with the subjects placed at different distances to achieve the specified loading levels. The lower exposure level falls below the Bowen threshold lung damage curve, while the higher load level falls above the threshold but below the 1% lethality curve.

Prior to blast exposure, and again 3 days or 1 month after blast exposure, macaques were evaluated using three cognitive tasks assessing different cognitive functions. Tissue examinations included magnetic resonance imaging (MRI), gross examination, light microscopy, and electron microscopy (SEM). Cellular level changes were mostly undetectable using MRI, though one macaque from the two-exposure, low overpressure group had a hyperintensive area in its cerebellum. Minimal lung damage was grossly observed, with a higher frequency and degree of subpleural ecchymoses and petechiae in the higher exposure group. However, blood gas analyses suggested that respiration and gaseous exchange were not significantly affected in any group.

In blast exposure groups, ultrastructural changes were observed in the brain tissue, and Purkinje neurons in the cerebellum and pyramidal neurons in the hippocampus were most vulnerable. These observations were consistent with behavioral changes and changes in motor coordination and working memory of the affected monkeys. This experiment provided a clear demonstration of primary bTBI in non-human primates that was observable in histological and behavioral assessments, but not in gross observations or MRI. However, specific biomechanical mechanisms contributing to the brain injury are not elucidated by this study due to whole body blast exposure.

## Overview of Biomechanical Mechanisms for Primary bTBI

How does a blast wave reach the brain to cause injury without external wounding? Current hypotheses can be grouped into three broad mechanisms, which are not mutually exclusive (Table 1). Each of these broad mechanisms and related experimental evidence will be discussed in some detail. These mechanisms may have different injury thresholds, which may help prioritize preventive efforts once the thresholds are quantified. First (in no particular order), since TBI has been repeatedly demonstrated to result from head accelerations that exceed certain thresholds in the context of blunt trauma, hypothetical mechanisms for acceleration-induced primary bTBI have also been suggested. Second, blast waves applied directly to the head might be transmitted through and reflected within the skull with sufficient magnitude to result in brain injury. Diverse specific mechanisms for direct cranial bTBI have been proposed. Third, thoracic mechanisms have been proposed whereby pressure waves originating in the thorax reach the brain with injurious magnitude. Specific hypotheses for a thoracic mechanism of bTBI include the initiation of bulk motion, resulting in a pressure surge in the vasculature, direct wave propagation via soft tissue or vascular structures, and/or a vaso-vagal neural response that may at least mediate the physiological response to blast exposure.

**Table 1:** **Brief summary of mechanisms of primary bTBI and selected relevant literature (due to space limitations)**.

**Acceleration mechanism**	
Translational and/or rotational accelerations of the brain caused by exposure to a blast wave may result in bTBI	
**Supporting**	**Confounding**

Head accelerations due to blunt force trauma are well documented to result in TBI; injury thresholds for such exposures have been published (23–25)	Injury thresholds are based on durations of acceleration significantly longer than accelerations induced by blast waves; extrapolation of injury tolerance curves may result in incorrect estimates
Experiments with rodents suggest intracranial pressures are lower when the head is restrained than when it is not when exposed to blast (26)	It is difficult to isolate an acceleration mechanism from direct cranial transmission in blast experiments
**Direct cranial entry**	
A pressure transient traverses the skull and directly injures brain tissue. The pressure transient may result from direct transmission and/or initiation of a pressure wave due to bulk motion of the skull. Localized peak pressures may result from constructive interference of internally reflected waves	
**Supporting**	**Confounding**

Numerical models suggest direct transmission and bulk motion; most predict at least localized intracranial magnification of external peak pressure^a^	Models are rarely calibrated with experimental data, are not quantitatively validated, and do not seem consistent with each other or with clinical incidence of bTBI
Experiments in rodents demonstrated a direct transmission mechanism (27, 28). Limited data support a contribution of bulk motion (29)	Thin rodent skulls provide little or no attenuation and so may not inform tolerance curves for humans. However, rodent models may be useful for investigating biomarkers and therapies
A few experiments using cadaveric heads measured transmission of externally applied simulated blast waves (30–32)	Results were highly variable between specimens. Inconsistent reporting of pressure prevents estimation of a transfer function. Results from exposure levels below injury threshold may not be as useful
Attenuation through pig skulls up to a factor of 8.4 may inform an upper bound for injurious exposure (33)	Pig skulls seem to provide more attenuation compared to the available cadaveric data
**Thoracic mechanism**	
Some combination of a pressure transient reaching the brain via the thorax and a vagally mediated reflex result in bTBI (14). Transient increased pressures in the cerebral vasculature may result from high speed propagation of a pressure transient without significant bulk motion and/or a later, slower volumetric blood surge	
**Supporting**	**Confounding**

Capillary hemorrhages in the brain resulted from single, fatal gunshot wounds to the thorax (34)	Some experiments of blast exposure to the thorax of animal models may include an unquantifiable direct cranial exposure (13) – and vice versa
Ballistic pressure waves in the thigh of pigs propagated to the brain via the vasculature near the speed of sound (35, 36)	A later volumetric surge cannot be ruled out from the reported results
Reviews of independent experiments in ballistics and behind-armor blunt trauma shows that pressure waves initiated in the thorax can cause cerebral effects (17, 37)	Physiological responses are mediated by the vagus nerve (13, 38); however, EEG signals were immediately suppressed even in vagotomized animals (39)
Mice with heads protected showed brain damage after thoracic exposure to blast (8)	Two phases of injury were apparent acute and longer over several days (inflammatory response).

*^a^Fuller discussions and additional references are in the text and cited review papers*.

A few additional mechanisms for primary bTBI have been proposed that do not fit easily into one of these three categories. For example, one hypothesis is that the piezoelectric properties of bone generate short range electric fields when exposed to blast (40, 41). Electric fields of certain magnitudes have known neurological effects. At this time, there is a lack of published data regarding this hypothesis.

## Acceleration Mechanism

It is well known that head accelerations due to blunt force trauma can cause TBI. Efforts have proven fruitful that correlate observed injuries with metrics based on translational and rotational accelerations applied over a specific time interval (23–25). Several such studies were recently reviewed and tabulated by Ganpule (31). Events related to automobile accidents, sports concussions, and falls tend to have interaction times longer than 3 ms, and the metrics associated with these studies are believed to have validity for accelerations with durations between 3 and 15 ms. The data on which the Ono curves (23) are based include shorter durations – down to 1 ms. Accelerations due to blast exposure tend to be of shorter duration than those due to blunt force impacts, and care should be taken when applying head injury criteria at durations shorter than the data on which they were originally based (42).

There is little doubt that at some threshold, head accelerations due to blast exposure cause TBI. However, if the injury threshold for the acceleration mechanism is much higher than for the thoracic and/or direct cranial mechanisms, then for practical purposes, it is less significant to bTBI. Conversely, if the injury threshold for the acceleration mechanism is much lower than for other mechanisms, then it would be expected to dominate for a certain range of exposures. Available data suggest that all three mechanisms have pressure thresholds in the neighborhood of 100 kPa for blast durations between 1 and 10 ms (18, 22).

Much has been written about which metrics are the best predictors of TBI at durations of acceleration common to impact (3–15 ms), whether translational or rotational accelerations are more important, and what scalings of injury thresholds are most appropriate between different animals (25, 43, 44). It is straightforward to apply existing criteria for acceleration-induced TBI to computational or surrogate models (42). It is much harder to test which injury predictors are likely to be valid. Several experimental techniques are available for isolating the cranial transmission and thoracic mechanisms of blast TBI; however, there is very little experimental work exposing animal models to injurious accelerations with durations from 0.3 to 3 ms without the confounding factors of other injury mechanisms.

Computational models are available and have been well-validated for blunt force trauma at durations above 3 ms. The head injury criterion (HIC), based on the Wayne State University tolerance curves for head injury due to blunt impact, has been widely used to study the risk of TBI due to blunt force trauma in automotive, sports, and other settings (45). However, experimental data used in its development did not include shorter durations of acceleration typical of blast wave exposures. Therefore, using that tolerance curve to estimate a threshold for acceleration-induced TBI would require extrapolation. The most extensive data in humans are available for the helmeted head in collisions between players of American football (25). The typical helmet to helmet contact time, the duration over which potentially injurious accelerations were being applied, was observed to be about 15 ms (46).

**Figure 1:**
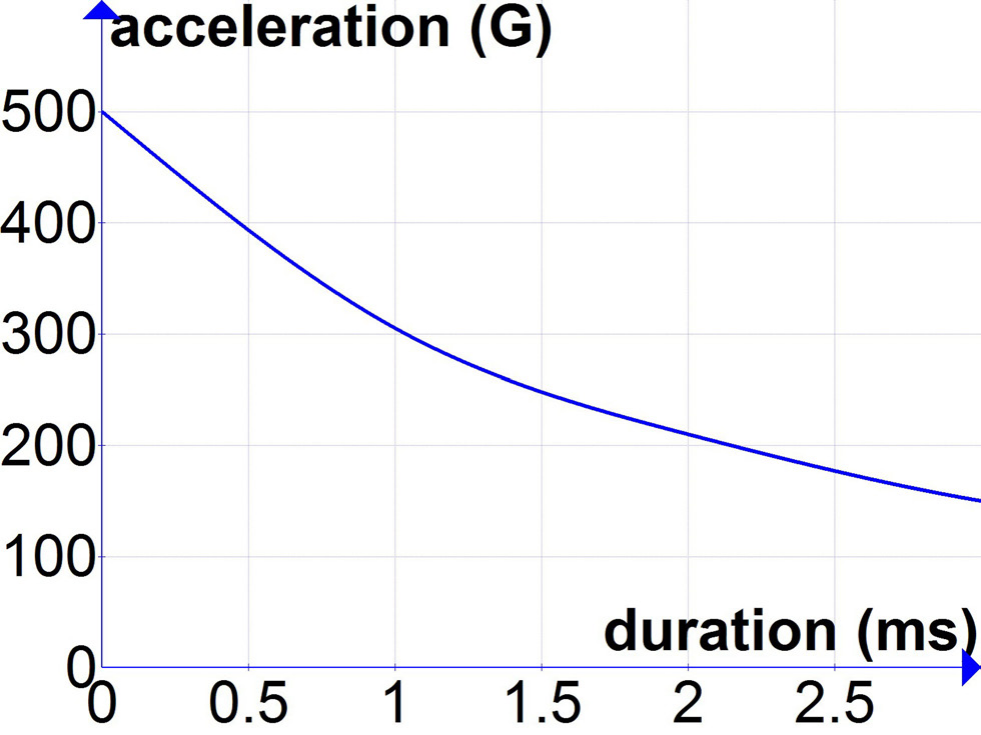
**Extrapolation of Ono curves (23) for durations of acceleration below 1 ms**.

New experiments are needed to better inform the threshold for acceleration-induced TBI for durations of acceleration of 0.5–3 ms that are common in blast exposures. Until new data are available, the best available approximations may come from the tolerance curve developed by Ono et al. (23). Experimentally induced rotational head accelerations used for the development of the Ono curve included durations ranging from 0.5 to 5 ms, and the published tolerance curve is illustrated for durations as short as 1 ms. Thus, using the Ono curve to estimate a threshold for acceleration-induced TBI due to primary blast exposure requires minimal extrapolation. One such estimate based on a linear extrapolation of the Ono curve for durations of <1 ms is shown in Figure 1.

An estimate for peak blast pressures associated with these accelerations was recently proposed and falls below the lung injury threshold for durations of 0.1–10 ms (18, 37).

## Direct Cranial Transmission

The hypothesis of direct cranial transmission suggests that a pressure transient traverses the skull into the brain and directly injures brain tissue. This mechanism is distinct from the mechanism of a coup-contre coup type injury that results from the brain contacting the skull, e.g., secondary to inertial effects after blunt trauma. When a blast wave reaches a boundary between two media (such as the air and the skin or underlying skull), a portion of the energy is reflected, a portion is absorbed by or scattered within the medium (e.g., the cranium), and a portion is transmitted (e.g., into the brain tissue). Some have estimated that transmission of blast waves is governed by the acoustic impedances of the materials on either side of a boundary (47). Acoustic impedance is defined as the ratio of acoustic pressure to flow (48). Acoustic impedance is used to compute, among other phenomena, the reflection and transmission of sound waves at the boundary of two media. The acoustic impedance model requires inputs of the speeds of sound in the respective media. The acoustic impedance of biological tissues has been of interest for many years and applied in technologies, including ultrasound imaging and lithotripsy (49). However, the acoustic impedance model of blast wave transmission assumes semi-infinite volumes of material and neglects effects of bulk motion. This helps to explain why results of experiments on blast transmission through layers of different materials often differ from theory based on acoustic impedances (50, 51).

Several numerical models developed to characterize the transmission of blast pressure waves into the human cranium have predicted results that do not agree with each other, though most predict magnification of an externally incident blast wave by 1.5–5 times at specific locations in the head or head surrogate [e.g., Ref. (52, 53)]. Alley (54) measured magnification of blast waves by two to five times at an anterior location in an instrumented, gel-filled polymethylmethacrylate (PMMA) sphere. By contrast, another finite element model predicted that peak intracranial pressure would be approximately equal to the peak pressure of the external blast wave; when the model included the meninges, peak intracranial pressure was only half that of the external blast wave (55). These widely differing predictions suggest that better validation is needed.

Ganpule (31) subjected three cadaveric heads (with meninges and brain tissue removed, substituted with ballistic gel and plugged distally) to a blast wave from a 28″ diameter shock tube with incident pressures of 70, 140, and 200 kPa (reflected pressure of about 600 kPa at the forehead) and duration of about 5 ms. The highest intracranial pressures were recorded closest to the incident wave (just behind the frontal bone) at 400 kPa – about two-thirds of the reflected pressure on the forehead; peak pressures behind the sinuses reached about 300 kPa and in the center and posterior locations peak pressures were about 150 and 75 kPa, respectively^6^. Despite similar preparation and blast exposures, peak pressures varied by up to 50% among the three specimens for the same sensor location, and impulse (the area under the pressure-time curve) varied up to 77%. Zhang et al. (32) subjected two post-mortem human head specimens (inverted to contain the natural brain tissues) to a blast wave from a 30″ diameter shock tube (at the specimen location) with incident pressures of 71, 76, and 104 kPa and duration of 6–7 ms. Pressures measured closest to the incident wave (just behind the frontal bone) were 1.5–2 times the incident pressure (sensor type was not specified). In these experiments, too, there were large differences in intracranial pressure–time data measured for similar exposures on the two specimens.

Some of these models and surrogate experiments suggest that peak intracranial pressures resulting from foreseeable blast exposures could be much higher than 100 kPa, which has been repeatedly shown to result in neural injury in direct impact models of TBI, such as fluid percussion experiments (56, 57). Results of fluid percussion experiments in animal models suggest that about 100 kPa is injurious to neural tissue, and that 200 kPa peak pressure on brain tissue may cause immediate incapacitation. Recall, however, that the vast majority of diagnosed cases of TBI in the U.S. Military from 2000 to 2014 are classified as mild level of injury (2). Based on these tissue level injury thresholds, the far greater number of diagnoses of mild bTBI, compared to moderate or serious TBI or death, does not seem consistent with estimates of peak pressures predicted by recent numerical models. However, as previously mentioned, data on the distribution of actual exposures to military personnel are lacking. Moreover, it is unclear how strongly the tissue injury threshold depends on the duration of exposure. Pressure pulses applied during fluid percussion experiments typically have durations of 15–20 ms.

Could the inconsistency between what might be expected from predictions based on numerical models and epidemiological data be explained by soldiers’ use of helmets? Probably not, because results of several numerical models and experiments with head surrogates (32, 58–61) suggest that, at least for helmets with suspension systems, a blast wave propagates in the space between the head and the helmet, and constructive interference on the opposite side results in magnified pressures on the skull.

Though model predictions seem inconsistent with epidemiological data, in principle, a direct cranial mechanism for bTBI seems intuitive. On the other hand, limited experimental evidence suggests that bone is an effective attenuator of blast waves. For example, Harvey and McMillen (62) reported results of experiments in which various tissue specimens were submerged in water whose surface was then impacted with a 0.125 caliber steel sphere (weighing 130 mg) at 3000 ft/s. Images recorded using the spark shadowgram technique showed that when the shock wave initiated by the impact interacted with a human cranium, a portion of the shock wave reflected, and a portion transmitted through the skull. Because the difference in acoustic impedance between air and bone is much larger than between water and bone, it is reasonable to expect that an even greater proportion of the shock wave would be reflected if the external medium were air rather than water. When a shock wave interacted with a slab of beef ribs submerged in water, the rib bones reflected the shock wave entirely, while the spaces between the ribs permitted transmission, accompanied by diffraction.

As mentioned above, the degree to which a blast wave is transmitted or reflected at a boundary is thought to depend on acoustic impedances of the adjacent materials, though current theoretical models are not quantitatively accurate (51). The acoustic impedance of the cranium is much greater than that of air. So on a material level, one might expect a small fraction of a blast wave to be transmitted through several millimeters of cranial bone. This was supported by a recent study in which deer skulls were exposed to blast waves with peak reflected pressure of 500–600 kPa and positive duration of about 2 ms. The peak reflected pressure of the transmitted wave retained only a fraction of the incident peak pressure (63). However, as the area over which the blast wave was applied increased, the transmission also increased, and additional features of the transmitted pressure wave suggested that bulk motion may have added to the transmitted pressure, as suggested by Moss et al. (58) and reported in a rodent model by Bolander et al. (29).

The exposure threshold for a direct cranial mechanism of bTBI in humans is not known. In a recent mouse model of blast injury, whole body exposure to 183 kPa peak pressure (measured with the sensor face parallel to the blast wave) generally resulted in mild injury (5% mortality) (64). Results in a rat model of blast injury in which the heads were exposed to about 240 kPa peak incident pressure resulted in “acute and enduring axonal injury, particularly in the cerebellum and brainstem,” along with an increase in the permeability of the blood–brain barrier (BBB) in the cortex (25% mortality) (65). Skopin (66) reported that disruption of the BBB caused similar deficits in spatial memory (as measured by the Barnes maze) compared to impact models of TBI in rodents.

Yeoh et al. (67) measured BBB disruption, mainly in the basal ganglia, in a rat model of bTBI due to localized cranial exposure to a very short duration (14–56 μs) blast wave with peak incident pressure of 145, 232, or 323 kPa. The 150-kPa group did not have significantly more lesions compared to the control group, while the 230 and 320 kPa groups did. These results suggested an injury threshold between 150 and 230 kPa under these loading conditions. Note the higher apparent tolerance at the shorter duration. Despite repeatable exposure characteristics, significant variability was observed between animals.

### Measurements of Intracranial Pressures in Various Models

Measurements in rats and mice exposed to whole body blast suggest that intracranial pressures are about the same as the externally applied pressure (26–28, 68, 69). Applied pressures in studies mentioned above exceeded those documented to result in brain tissue injury in fluid percussion experiments. During the blast experiments, however, rodents were anesthetized, so the effects of blast exposure on incapacitation could not be observed for comparison.

In addition to providing experimental evidence of primary bTBI, these rodent models provide an initial estimate for intracranial pressures that may result in bTBI in humans as well as a model for testing therapies. The rat cranium has been reported to be <1 mm thick, and in contrast to the human skull it does not contain a dipole (trabecular-like) layer between inner and outer tables of cortical bone in some regions. Therefore, the rat cranium may represent an upper bound for the transmission of blast waves through cranial bone, so that injury thresholds determined from data in rodent models may represent a lower bound for human exposures.

Recent investigations have helped to quantify what pressure levels at a given duration are injurious in rodent models of primary bTBI, without focusing on a specific mechanism. Zhu et al. (70) used a combination of analytical considerations and experimental results from eight studies of blast exposure in rats (some cited herein, plus others) to estimate an injury risk curve for primary bTBI in the rat. Their results suggested that a 2-ms exposure to about 100 kPa peak incident overpressure is the brain injury threshold for bTBI in the rat, and a 2-ms exposure to 200 kPa was associated with a 50% risk. This injury threshold of 100 kPa is consistent with previous estimates of the brain tissue injury threshold and the reports that blast waves traverse the rodent cranium with little if any attenuation, as discussed above. The confidence in the injury risk curves estimated by Zhu et al. is limited due in part to the different sensitivities to detect injury used in the various experiments. Moreover, the authors pointed out that the assumption that the risk curves for bTBI parallel Bowen’s injury curves for the lung was for expediency and needs experimental support or correction.

Shridharani et al. (33) reported intracranial pressure measurements from anesthetized pigs (average mass 61 kg) whose heads were exposed to blast waves with peak reflected pressures of 110–740 kPa and durations (based on a scaling factor) of 1.3–6.9 ms. The peak intracranial pressure was attenuated up to a factor of 8.4. The transmission of a blast wave through a porcine cranium may represent a lower bound for transmission through a human skull, so that injury thresholds determined in a similar model may represent an upper bound for humans.

Bir (30) reported results of experiments in which a compression-driven shock tube was used to apply blast waves to four cadaveric heads, and surface strains as well as intracranial pressures were measured at several locations for different orientations of blast exposure. Each head was subjected to five exposures at three levels of peak incident pressure, 69, 88, and 120 kPa. Pulse durations were 7–8 ms. Peak intracranial pressures varied with location but had a maximum value approximately 1.7 times the externally applied incident pressure (corresponding to about half of the reflected pressure) at some location. Both tensile and compressive strains were measured on the surface of the skull at each location; magnitude and the relative time courses of tensile and compressive strains varied with orientation with respect to the blast wave. For the highest load level used (137.9 kPa, measured with the sensor face parallel to the blast wave), peak measured tensile and compressive strains were about 0.075 and 0.05%, respectively. These levels of strain are not expected to result in microdamage to the bone based on observations at quasi-static strain rates. The first and fifth trials at each loading level (trials 1 and 5, 6 and 10, 11 and 15) were repeat measurements of a frontal exposure; results at each loading level were consistent. Both the surface bone strain levels and the consistency of the intracranial pressures with repeated loading indicate that the transmission of the shock wave was not increasing with repeated loading at these levels. For further context, the highest loading level was below the threshold for lung injury due to blast exposure.

These efforts notwithstanding, investigations are needed to determine what level of pressure (at a given duration) is injurious to human brain tissue. A separate but essential area of investigation is to determine the transfer function for blast pressure from the outside to the inside of the human head. As illustrated by the available data from post-mortem human specimens, the challenges of determining blast wave transmission through the human head with accuracy or precision are many. It is not clear what simplifications (e.g., substitution of the meninges and brain tissue with ballistic gelatin), handling protocols (formaldehyde fixation, temperature), and other experimental details (placement of sensors, coupling of sensors to tissue) significantly affect the accurate assessment of blast wave transmission because the data are too sparse. Even within the same experimental protocol, specimen to specimen variations have differed by a factor of two (31, 32). It is anticipated that variations in skull size and thickness will contribute to individual differences in blast wave transmission, suggesting that a normalization scheme may be needed to develop injury risk curves – possibly based on simple geometric parameters (skull thickness at certain locations, cranial volume, surface to volume, etc.). However, the currently available data is far too sparse to support a normalization scheme at this time, and published research to date includes no parametric studies of the effects of boundary thickness on blast wave transmission in surrogate tests.

## Thoracic Mechanism

Several specific thoracic mechanisms of primary bTBI have been proposed. One hypothesis is that a blast wave applies pressure on the thorax to cause a volumetric blood surge, leading to an increase in intracranial blood pressure great enough to damage the BBB and capillaries in the brain from the inside out [as in Ref. (71)]. The neural damage is then hypothesized to result from exposure to extravasated blood products, edema, and hypoxia.

Another, distinct hypothesis for a thoracic mechanism of primary bTBI is based on the propagation of pressure waves from the thorax to the brain – perhaps via the soft tissues or the vasculature more specifically [as in Ref. (72)]. In general, stress and pressure wave propagation does not require bulk motion but is a movement of energy through a medium. The speed at which the energy travels depends on the medium. For example, in our perception of the everyday world, the faster speed of sound in water vs. air may be a familiar concept. Pressure and stress waves travel near or above the speed of sound.

Ballistic pressure waves also travel near or above the speed of sound, and remote wounding effects of ballistic pressure waves have been referenced since the nineteenth century. Rigorous experimental support has been published more recently [e.g., as reviewed by Courtney and Courtney (17), and references therein]. Briefly, several different research groups performed experiments using canine and porcine models and observed neural damage in the brain after penetrating ballistic insult to the animal’s thigh. For example, Suneson et al. (35) reported results of experiments on pigs (mean weight 21.5 kg) that were shot with a small steel sphere in the left thigh. The amplitude of the pressure in the abdomen was reported to be about 270 kPa, and in left frontoparietal region of the brain about 125 kPa. No macroscopic changes in the brain were observed (35). In a similar study, Suneson et al. (36) measured positive peak pressures in the brain of 150 kPa; in these experiments, a transducer was also placed in the right common carotid artery of several animals. They reported, “In all cases the [pressure] amplitudes were larger inside the skull than in the [one] artery.” Again, no macroscopic changes, such as hemorrhage or contusion, were observed. However; light microscopic studies showed some BBB damage and damage to larger axons; for animals maintained 48 h after the initial insult, glial changes, and edema were also reported. These changes were more severe in the cervical spinal cord and brainstem than in other brain regions. These results are consistent with those of similar studies in dogs, in which damage was observed in the hippocampus and extended to neurons in the hypothalamus and cerebellum for more severe insults [Ref. (17), and references therein]. Of note, Lai et al. (72) performed electron microscopy on the vasculature of dogs thus injured and observed damage to the vascular intima in the aorta, common carotid, and middle cerebral arteries. More recently, Krajsa (34) reported capillary hemorrhages in human brains at autopsy from 33 individuals who experienced a fatal penetrating chest wound by a single bullet. All other traumatic factors, including historical factors, were excluded during case selection process. The findings were attributed to “sudden changes of the intravascular blood pressure as a result of a compression of intrathoracic great vessels by a shock wave caused by a penetrating bullet.”

In the experiments conducted by Suneson et al. (35, 36), the pressure wave initiated by penetrating insult to the thigh propagated to the abdomen and brain close to the speed of sound. This suggests that a mechanism of wave propagation was present; however, the reported results do not rule out a later volumetric surge of blood.

Similar injuries to the central nervous system (in addition to lung damage) were documented after a blast pressure wave was applied to the thorax in a rabbit model (15). The results of that study and of a contemporaneous study by Irwin et al. (38) were consistent with a thoracic contribution to bTBI; however, questions were also raised. For example, the relative exposures to the thorax and the head were not known. By contrast, pressure waves in the thorax caused by penetrating ballistic projectiles or ballistic impacts to body armor can cause cerebral effects and can only reach the brain via an internal mechanism (17). Cernak et al. (13) and Irwin et al. (38) observed that aspects of the physiological response to blast were mediated by the vagus^7^ nerve. In a later series of experiments on swine exposed to non-penetrating ballistic impact to thoracic armor, vagotomy reduced apnea and bradycardia due to ballistic pressure waves (39, 73, 74). However, vagotomy did not eliminate neural effects in the brain, suggesting that the pressure wave directly affected the brain cells via a thoracic mechanism, which is further supported by the immediate suppression of EEG signals in some of these experiments.

There is a growing body of repeated experimental results showing BBB damage in the basilar and hippocampal regions following blast exposure in animal models (26). These results are consistent with results of diffuse tensor imaging in military personnel following blast exposure showing changes in the basilar region; however, while clinical studies have shown differences between blast-exposed and matched control groups on average, results are not sensitive or specific enough to be diagnostic [e.g., in Ref. (75)]. BBB disruption via a thoracic mechanism of bTBI does not exclude BBB disruption from a cranial mechanism of bTBI. As discussed above, Yeoh et al. (67) observed BBB disruption in a rat model of bTBI due to localized cranial exposure.

Cernak (7) reported results of a series of blast experiments on mice that were provided no protection, head protection, or thoracic protection from blast exposure. Whole body blast exposure to a peak pressure of about 180 kPa resulted in 5% mortality. In surviving animals, a multi-phase cellular response was observed over time using bioluminescence imaging. This multi-phase response was described as acute alterations followed by chronic alterations, such as inflammation, which can lead to irreversible degenerative changes. In animals with *torso* protection only, acute and chronic responses in the *brain* were significantly less than in animals with head protection only or that experienced whole body exposure. These results suggest that, for this level of exposure, thoracic, and other mechanisms contributed to the bTBI; the thoracic mechanism was a significant contributor to the overall injury, which was dramatically reduced by thoracic protection.

By contrast, some experimental results may be misinterpreted to discount the existence of a thoracic mechanism. Some experiments do not inform the issue one way or another because of their design. For example, measurements of intracranial pressures in isolated cadaver heads exposed to blast from a shock tube may elucidate a direct cranial mechanism of primary bTBI but cannot inform a thoracic mechanism [Ref. (31), pp. 30–31]. It is important to remember that mechanisms of bTBI are not mutually exclusive, though different injury thresholds, once demonstrated, may render one mechanism more practically relevant than another.

For another example, Goldstein et al. (26) sought to investigate acceleration, direct cranial, and thoracic mechanisms of primary bTBI in mice. Specifically, intracranial pressures and head accelerations were measured in mice exposed to a peak incident pressure of 77 kPa from a shock tube, with and without head immobilization. In addition, intracranial pressures were measured in isolated, reperfused mouse heads exposed to the same pressure profile. The contribution of head acceleration to intracranial pressure in this model was clearly demonstrated. However, the results were interpreted as showing that there was no thoracic contribution to the cranial pressure response. The interpretation was based on a premise that if a thoracic mechanism contributed to increased cranial pressures, then a delayed peak pressure in the cranium was expected in the intact animals compared to the isolated heads; in addition, a reduced peak pressure in the cranium might be expected in the isolated heads. The published data actually show an extra peak in pressure vs. time, approximately 1.5 ms after the initial peak, and this extra peak is absent from the measurement in isolated heads. In addition, the peak impulse (the area under the pressure-time curve) in intact mice was double compared to that in isolated heads. The interpretation of the results regarding a thoracic mechanism is not supported by the data presented in the figures.

The experimental evidence for a thoracic mechanism of primary bTBI is strong, having been demonstrated in various animal models under various conditions. An outstanding question is, what is the injury threshold (in terms of peak pressure and duration)? Courtney and Courtney (18) analyzed results of blast experiments and related studies, including behind-armor blunt trauma and ballistic pressure wave studies. The results were shown graphically as a region of interest for a thoracic mechanism of primary bTBI overlaid on the familiar Bowen plots for blast-induced lung injury. For blasts of duration 1–2 ms, the region of interest extended from 100 to 400 kPa; the lower boundary was shifted upward to 200 kPa for blasts of shorter duration. The result suggests that the threshold is below the lung injury threshold at durations expected for IED type threats (0.1–2 ms) and likely overlap with the injury threshold for an acceleration mechanism of bTBI for some exposures. In the absence of quantitative field data on exposures and outcomes, support or correction based on human-specific information is still lacking. The practical importance of a thoracic mechanism in humans depends on where injury thresholds lie relative to thresholds for blast lung damage as well as acceleration and direct cranial mechanisms of bTBI.

## Scaling Laws

Potentially, injurious blast experiments are necessarily restricted to animal or inanimate models. In some efforts to interpret results of blast experiments in animals to human exposures, scaling laws have been applied. A scaling law in this context is a mathematical transformation of data supposed to make the results more applicable to humans. For example, the body mass-based scaling for the Bowen curves for blast-induced lung injury was developed using experimental data from a wide range of animal species and sizes (76); this scaling fit the data well and extrapolation was not required to scale the results for humans. No scaling was used in the development of the regions of interest for a thoracic mechanism of bTBI discussed above. Perhaps with an appropriate scaling, more precise thresholds could be identified.

However, appropriate scaling laws to apply results of animal studies to human bTBI injury thresholds are not established. Different scaling may be appropriate for different mechanisms of bTBI. For example, Gibson (44) proposed a scaling rule for acceleration-induced TBI based on brain mass. Depending on the extent to which a thoracic mechanism of bTBI is related to chest wall acceleration, a body-mass scaling may be appropriate. Zhu et al. (70) proposed a scaling of bTBI tolerance curves from the rat to the pig and then to the human based both on body mass and a scaled duration of exposure. The resulting tolerance curves diverge as the duration of exposure decreases, with the tolerance in the pig and human predicted to be about 1.5 times that of the rat for a 1-ms exposure.

## Discussion

Given the resources expended to unravel blast injury mechanisms over the past decade, the lack of more definitive thresholds for each candidate mechanism is disappointing. Key aspects of sound scientific methodology have been neglected. Rather than design experiments for focused testing of explicit hypotheses regarding mechanistic and threshold questions, many experimental designs attempt to more broadly speak to an array of relevant questions and issues.

Overly optimistic claims of validation for computational models represent another departure from the scientific method and have hindered progress regarding mechanisms and thresholds of bTBI. It is dubious to claim validation for blast models using non-blast experiments at much lower strain rates and when the predicted pressures only agree with the measured pressures for a small fraction of the blast wave duration. Credible claims of validation should specify a percent difference between the predicted and measured physical values as well as the duration over which agreement remained within the specified percent difference. Then, different models can be more meaningfully compared and improvements quantified.

It is likely that unavailability of physical properties at blast strain rates is hampering the development and validation of truly predictive models. For example, numerical models of direct cranial transmission of blast waves utilize a relationship between elastic modulus and density for bone elements, often without considering viscoelastic effects. However, bone is a viscoelastic material, so the mechanical properties depend on the rate of loading. The strength of human bone has been measured to increase by 30% when the strain rate increased from 1 to 100 s^−1^ (77). In addition, above strain rates of 10 s^−1^, the presence of marrow, such as contained in the dipole layer of the skull, was reported to have an additional strengthening effect. The results suggested that “the presence of marrow during severe, traumatic, compressive loading *in vivo* may serve to absorb considerable energy” (78). In addition, viscoelastic damping in human cortical bone has been reported to vary by about an order of magnitude over loading frequencies from 1 to 10^6^ Hz (79). Whether these or different relationships apply at strain rates applied by blast waves, the effects on model predictions of using simplified relationships for bone mechanics is unknown. Brain tissue is highly viscoelastic as well as anisotropic (properties are different depending on the direction of loading). Attempts to quantify the viscoelasticity of brain tissue, even at lower strain rates, and in particular to experimentally verify constitutive relationships, has proven challenging (80–83).

Where possible, a fruitful approach moving forward would be to use the physical properties as adjustable parameters in blast experiments with very simple geometries with only a few materials to accurately determine values of material properties independently of more complex biofidelic geometries with many different materials. Once accurate material properties are obtained from gaining agreement with experiments and models in simple geometries, predictions can be made from running a full biofidelic model with accurate material properties at blast strain rates.

Reconstructions of actual human blast exposures can also be used to greater benefit in validating models and elucidating mechanisms. Helmet mounted sensor systems have been fielded since at least 2009 (59), so there should be some body of data available to compare exposures with resulting injuries. There is also the potential to utilize bodies of data related to human exposure to muzzle blasts of cannon (84), exposure of entry teams to breaching blasts (10, 85), and exposure of room occupants near flash grenades.

Ultimately, the emergence of confidence regarding mechanisms and thresholds of bTBI may depend on combining a knowledge base built from controlled experiments, validated modeling, and real-world exposures analogous to the approach of Zhang et al. (25). The challenge for contributors to this process is in designing good experiments, accurately assessing model validity, and identifying opportunities to compare model predictions with real-world blast exposures.

## Conflict of Interest Statement

The authors declare that the research was conducted in the absence of any commercial or financial relationships that could be construed as a potential conflict of interest.

## Footnotes

^1^For example, in 1915, the British army in France was instructed that if a soldier manifested “shell shock” following an actual shell explosion, he was entitled to be classified as “wounded” with the associated benefits, whereas, without known blast exposure, those displaying similar symptoms were classified as “sick” and were not entitled to the same recognition or benefits (Ben. A War of Nerves: Soldiers and Psychiatrists, 1914-1994. London, Jonathan Cape, 2000).

^2^The Defense and Veterans Brain Injury Center stated that the total represents medical diagnoses of mild, moderate, severe, and penetrating TBI (rather than self-report).

^3^Concussion/mild TBI was characterized by a confused or disoriented state lasting <24 h; loss of consciousness for up to 30 min; memory loss lasting <24 h; and structural brain imaging (MRI or CT scan) yielding normal results.

^4^Public Law 110-28, May 25, 2007

^5^NIH Estimates of Funding for Various Research, Condition, and Disease Categories (RCDC) http://report.nih.gov/categorical_spending.aspx accessed November 20, 2014.

^6^As the method is presented in Ganpule (31), it appears that tests were conducted in three repetitions each at each of the three peak pressure levels, increasing. Surface strains on the cranium were not reported to reach levels at which damage is expected to occur in cortical bone, based on quasi-static tests.

^7^The vagus nerve is Cranial Nerve X (10) and is also known as the pneumogastric nerve.
